# Trait emotional intelligence in American pilots

**DOI:** 10.1038/s41598-022-18868-4

**Published:** 2022-09-02

**Authors:** Zachary Dugger, K. V. Petrides, Nicole Carnegie, Bernadette McCrory

**Affiliations:** 1 Department of Mathematical Sciences, United States Military Academy, West Point, NY USA; 2 London Psychometric Laboratory, University College London, London, UK; 3 Department of Mathematical Sciences, Montana State University, Bozeman, MT USA; 4 Department of Mechanical and Industrial Engineering, Montana State University, Bozeman, MT USA

**Keywords:** Psychology, Human behaviour

## Abstract

There is a dearth of trait emotional intelligence (trait EI) research within an aviation context. Using the Trait Emotional Intelligence Questionnaire (TEIQue), the present study investigated potential trait EI differences between pilots and general population controls in the United States. The forty-four pilots who volunteered to participate were primarily male (93%) and between 24 and 67 years with a wide range of flight experience (150–5000 + hrs.) They were matched with controls based on age, gender, and ethnicity. Comparisons on global trait EI and the four trait EI factors revealed significant differences, with pilots scoring consistently lower than their matched counterparts in *global trait EI*, *Well-being*, *Emotionality*, and *Sociability,* but not *Self-control.* Overall, the findings indicated that pilots felt less connected to their emotional world than controls. Though limited by sample size and participant diversity, the results provide a basis for future studies into the trait EI profile of pilots, which had not been previously investigated.

## Introduction

Trait emotional intelligence can be defined as a constellation of emotional perceptions assessed through questionnaires and rating scales^1^. The theory focuses on emotional perceptions and presents a robust operationalization framework, particularly psychometrically^2^. The trait EI model is salient in the scientific literature being the focus of hundreds of peer-reviewed publications (for a review, see^3^ and dedicated special issues^4^. Since its inception, the construct has been strongly linked to behaviors that constitute key components of the pilot skillset, including leadership^5^, mental toughness^6^, and stress management^7^.

Despite its increasing prominence, little research has focused on trait EI within an aviation context. The isolated studies that have been carried out so far have found that the construct is positively associated with pilot training performance among military flight students in the United Kingdom^8^ and self-reported safety citizenship behaviors in American military pilots^9^. However, in both these studies, only military pilots were evaluated, and no comparisons were conducted to explore differences between pilots and the general population. In stark contrast, personality traits have long been evaluated within aviation research and found to be linked to crew coordination^10^, training success^11^, and pilot selection^12^. Pilots appear to have high extraversion scores (particularly evident in facets such as assertiveness, activity, and excitement seeking) when compared to population norms^13^. Pilots also score lower on neuroticism than non-pilot peers, indicating an ability to handle fear, anxiety and stress^14,15^.

The operation of complex aircraft often involves multiple crewmembers^10^. Pilots require teamwork skills, mental and emotional fortitude, as well as adaptability to operate as a cohesive team over prolonged flights, collaborating to accomplish tasks in complex and stressful environments^16^. Accomplished pilots must be able to manage emotions, assess risk accurately, and work together with other crewmembers throughout the flight. Therefore, trait EI likely plays a key role in pilots’ ability to operate as crewmembers.

The present study investigated possible differences in trait EI between pilots and the general population in the United States. Given the dearth of trait EI research within aviation and the close links between trait EI and personality^1,3^, our research design was modelled on previous studies into the personality traits of pilots (e.g.,^13–15^). Participants were evaluated using a standardized psychometric instrument (Trait Emotional Intelligence Questionnaire; TEIQue), allowing for a statistical comparison of two distinct groups: pilots vs non-pilot controls.

While the study was exploratory in principle, motivated by similar research that previously examined personality differences among different occupational groups, we did formulate two specific hypotheses at the factor level of trait EI:

**H1** Pilots will score higher on Self-control than non-pilot peers. Pilot personality has been linked to emotional stability. Gao and Kong^15^ conducted a study of personality types among Australian college students and found that pilot students in that sample had lower Neuroticism scores than non-pilot peers, a noticeable benefit for a role often requiring the ability to manage high stress effectively. Additionally, a recent neurological study indicated that the neural mechanisms for handling emotional conflict differ between pilots and non-pilots with the former showing higher emotional stability than the general population, as might be expected from a profession that requires affective control and focus^17^.

**H2** Pilots will score lower on Emotionality than non-pilot peers. This hypothesis stems from the pilot job specification and masculine culture, which urge emotional stability and deter emotional openness and vulnerability^18,19^. Aviation culture, both military and civilian, encourages more masculine traits among pilots^20^ and, therefore produces pilots less inclined to externalize overtly emotional behavior.

## Methods

### Participants

Volunteer pilots from numerous aviation organizations within the United States were contacted, forty-four (44) of whom agreed to participate in this Institutional Review Board-approved study. All participants were required to hold a currently valid flight qualification (i.e. FAA certificate or military rating). They were between 24 and 67 years old $(\overline{x }=34.6, s=8.87)$ and had a wide range of flight experience (150–5000 + hrs). The cross-sectional sample included both fixed-wing (n = 29) and rotary-wing (n = 15) pilots. Participants were primarily male (93.2.%), white (90.9%), and had completed at least some college (97.7%). Of those, 93.2% were either currently serving, or had previously served, as a military aircrew member. Eligibility for the survey was based on current flight status and pilot qualification (i.e. FAA license and/or military qualification). Pilots were recruited based upon multi-pilot aircrew experience; having served with other aircrew members in a collaborative role. Pilots from multiple aviation organizations (military and civilian) volunteered to participate and all participants signed an informed consent prior to completing the survey.

The control group was drawn from a TEIQue US dataset (n = 531) and was matched on age (±3 yrs), gender, and ethnicity, which are factors known to affect trait EI^21,22^. Additionally, participants were matched, where possible (73%), by educational level across seven educational levels (±1 level), ranging from High School Diploma (1) to Doctoral Degree (7) in order to reduce possible confounding due to education differences^23,24^. The control sample was matched to the pilot sample at a 2:1 ratio, resulting in 88 non-pilot control subjects for a total sample of 132 participants. Additional factors of interest were English fluency and employment (Table 1).

**Table 1:** Matching Criteria and Pertinent Participant Demographics.

	Characteristic	Proportion (%)	
		Pilot	Control
Age^M^	20–29	29.5	27.3
	30–39	52.3	54.5
	40–49	11.4	11.4
	50–59	2.3	2.3
	60–69	4.5	4.5
Gender^M^	Male	93.2	93.2
	Female	6.8	6.8
Ethnicity^M^	White	90.9	90.9
	Other^1^	9.1	9.1
Education Level^M^	Associate’s Degree or below	31.8	29.5
	Bachelor’s Degree	59.1	37.5
	Graduate Degree	9.1	33.0
Employment Type	Full-time	95.5	86.4
	Part-time	–	2.3
	Student	4.5	6.8
	Other^2^	–	4.5
English Fluency	Completely articulate	100	93.2
	Mostly articulate	–	6.8

^M^Matching Criteria.

^1^Includes persons of Black/African American, Hispanic/Latin/Spanish, and Mixed Race/Ethnicity.

^2^Includes unemployed and unidentified employment status.

### Measures

Trait EI was measured with the Trait Emotional Intelligence Questionnaire (TEIQue v. 1.50;^22^. This is a 153-item inventory covering the four factors and 15 facets that comprise trait EI (Table 2). It has demonstrated satisfactory psychometric properties in numerous studies, languages, and cultures (e.g.^22,25–28^). Items are responded to on a 7-point Likert-type scale, ranging from “1 = Completely Disagree” to “7 = Completely Agree.” Internal reliability analysis indicated robust alpha values (> 0.70) for global trait EI as well as for all four factors across both groups (pilots and controls).

**Table 2:** Trait EI definitions and descriptive statistics broken down by group (pilot vs control).

Factors/facets	High scorers perceive themselves as…	Pilots (n = 44)		Controls (n = 88)	
		Mean (SD)	α	Mean (SD)	α
**Well-being**		5.24 (0.47)	0.77	5.94 (0.54)	0.73
Self esteem	… confident with a positive view of themselves				
Happiness	… cheerful and satisfied with themselves				
Optimism	… likely to “look on the bright side.”				
**Self-control**		5.09 (0.65)	0.75	5.25 (0.71)	0.76
Emotion control	… able to control their own emotions				
Stress management	… able to regulate stress and withstand pressure				
Impulse control	… reflective and less likely to give in to urges				
**Emotionality**		5.17 (0.70)	0.84	5.52 (0.59)	0.78
Emotion perception	… clear about their own and others’ feelings				
Emotion expression	… fluent in communicating their emotions to others				
Relationships	… capable of creating fulfilling personal relationships				
Empathy	… able to take someone else’s perspective				
**Sociability**		5.16 (0.50)	0.80	5.38 (0.57)	0.75
Social awareness	… networkers with excellent social skills				
Emotion management	… able to influence others’ feelings				
Assertiveness	… forthright and frank, willing to stand up for their rights and beliefs				
Adaptability*	… flexible and willing to adapt to new environments and conditions				
Self-motivation*	… driven, determined, and able to persevere				
Global trait EI		5.70 (0.56)	0.90	5.49 (0.45)	0.88

SD = Standard deviation.

α = Cronbach’s alpha measure of internal consistency.

*Denotes facets unrelated to four trait EI factors. Both facets are accounted for though global trait EI score.

### Analyses

Initial data screening was conducted in accordance with the TEIQue guidelines. One case was discarded due to multiple omitted answers (> 15%). Statistical analyses were performed using the SAS software (Version 9.4, SAS Institute, Inc, Cary, North Carolina). The level of significance was set at α = 0.05 (two-tailed). Bonferroni adjustments were applied to ensure the 95% confidence intervals for the estimates matched significance levels. General linear regression models were used to estimate the effects of study group (pilot vs control) on global trait EI and its four factors: Well-being, Self-control, Emotionality, and Sociability. Ethnicity was dichotomized as white (0) versus all other ethnicities (1) and education level was trichotomized as associate degree or below (0), bachelor’s degree (1), and graduate degrees (2). Since age, gender, ethnicity, and education were included in the matching process, interactions with these variables were not considered. There were no trait EI differences based on flight experience (p = 0.47) and this variable was excluded from further analysis.

### Ethics

This study was approved by the Institutional Review Board at Montana State University (IRB #ZD071720-EX) as an exempt research protocol CFR, Part 46, Section 101 (b)(2). All participants provided written informed consent prior to participating, and all data were deidentified. All research methods were conducted in accordance with relevant guidelines and regulations.

## Results

After controlling for age, gender, ethnicity, and education, the general linear model indicated significant group differences in global trait EI scores [F (1,125) = 7.90; p = 0.006], Well-being [F (1,125) = 6.33; p = 0.0132], Emotionality [F (1,125) = 6.16; p = 0.0144], and Sociability [F (1, 125) = 6.16; p = 0.0144]. In contrast, no significant differences were identified in Self-control scores [F (1, 125) = 1.04; p = 0.309]. Table 3 presents the details of this analysis. Pilots exhibited lower least squared mean scores in global trait EI, Well-being, Emotionality, Sociability, but not in Self-control.

**Table 3:** Pilot vs control group comparisons on the TEIQue.

Measure	LS Mean	SE	F statistic (df)	Bonferroni t-value	p-value
**Global trait EI**					
Pilot (n = 44)	5.44	(0.12)	F (1, 125) = 7.90	t_125_ = 2.81	0.006**
Control (n = 88)	5.69	(0.11)			
**Well-being**					
Pilot (n = 44)	5.99	(0.14)	F (1,125) = 6.33	t_125_ = 2.52	0.0132*
Control (n = 88)	6.24	(0.12)			
**Self-control**					
Pilot (n = 44)	5.37	(0.17)	F (1,125) = 1.04	t_125_ = 31.58	0.3088
Control (n = 88)	5.49	(0.15)			
**Emotionality**					
Pilot (n = 44)	5.44	(0.17)	F (1,125) = 6.16	t_125_ = 2.48	0.0144*
Control (n = 88)	5.74	(0.15)			
**Sociability**					
Pilot (n = 44)	5.05	(0.17)	F (1, 125) = 6.16	t_125_ = 2.48	0.0144*
Control (n = 88)	5.34	(0.14)			

*p < 0.05; **p < 0.01.

## Discussion

The present exploratory study investigated potential trait EI differences between pilots and the general US population. The results provided support for the second hypothesis, but not the first. More specifically, pilots scored lower than control counterparts on global trait EI as well as on three of the construct’s four factors: Well-being, Emotionality (H2), and Sociability. However, they did not score higher on Self-control (H1) as had been hypothesized. The common core of the factors of Well-being, Emotionality, and Sociability concerns a positive assessment of one’s emotional capabilities, which often extends into areas beyond emotion and, in its maladaptive manifestation, veers into narcissism and hubris^29^. It is, therefore, not surprising that pilots, who need to be careful, straightforward, and understated in their work have modest TEIQue scores compared to peers pursuing goals that may well engender or even require self-promotional and narcissistic attitudes, such as advancement in formal organizational hierarchies^30^. Similarly modest trait EI scores have been observed with military managers, who are also required to keep narcissistic tendencies well in check^31^.

Another reason for lower trait EI scores in pilots relates to organizational culture. Pilots have long been associated with a masculine culture that emphasizes aggressiveness, competition, and performance orientation^20,32^. This aligns with H2 as, in effect, pilots are in one of those organizational cultures that have not yet experienced the “Affective Revolution”^33^ and thus remain unenthusiastic about feelings, preferring an ethos that underscores the perception of invulnerability and resistance to human weaknesses^34^. In practice, the pilot selection and training process may produce pilots, primarily male but also female, who fit within this culture. Longitudinal analysis of pilot trait EI scores over the duration of their training and early career years may yield important insights into the effects of organizational culture on pilot trait EI.

The results did not bear out H1, as there was no difference between pilots and controls in the Self-control factor of trait EI. Self-control has been linked to the ability to maintain situational awareness and control^35–37^. Despite the null result, it is worth noting that Self-control was the only trait EI factor in this study where pilots did not score significantly below their non-pilot counterparts.

## Limitations and Future Research

The present study was conducted with a relatively small sample of American pilots (n = 44). Accordingly, the analysis and conclusions were confined to the factor level of trait EI without delving into the 15 constituent facets. Analyses at the facet level will require further investigation on a larger sample size, but may yield significant insights into potential interactions between facets, a possibility that could not be explored in this study. Without doubt, further research is required to establish the complete trait EI profile of pilots, research that will have to be conducted at the facet level of the construct.

The control sample used in this study predominantly comprised persons with higher education than the pilot sample. For this reason, we were only able to match 73% of the pilots with a control from a comparable educational level. A larger, more educationally diverse control group would have enabled a more exact matching process. Additionally, this study was conducted with a group of pilots that were predominantly White males with at least some military background. Military pilots tend to undergo more rigorous training than their civilian counterparts^38^ and serve within a distinct organizational culture^39^. For these reasons, the findings may not fully generalize to the entire international aviation industry. Future studies should seek to incorporate a more diverse sample of pilots, considering such background variables as gender, ethnicity, educational level, military background, and prior experience.

Understanding the prevalent trait EI profiles of pilots has the potential to inform future pilot selection processes by helping to identify potential candidates with the characteristics that are predictive not only of performance per se, but also of organizational and cultural fit. Parallel initiatives can be incorporated into training and development programs targeting current incumbents. The utility of personality inventories as selection tools has been discussed and corroborated consistently within aviation (e.g.^40–42^), but the emotion-related aspects of personality remain seriously and inexplicably underexplored. Further study of the relationships between trait EI and successful pilot performance, targeted at identifying the specific factors and facets that are most pertinent to pilots is now warranted. The present findings indicate that pilots tend towards neutral trait EI scores. The full repercussions of this in relation to such outcomes as job performance, satisfaction, and commitment ought to be systematically determined.

The cross-sectional nature of our research design precluded an assessment of any impact of introductory training and organizational culture on pilot trait EI profiles. Research has indicated that personality, which is considered a generally stable characteristic and plays an important role in career decision-making, may be altered through intense training or social experiences^43^. Longitudinal analyses have the potential to deliver key insights into the interrelationships between training, culture, performance, and their dynamic interaction with trait EI profiles.

## Conclusions

The goal of this study was to investigate potential differences in the trait EI profiles of pilots and the general population in the United States. Overall, the findings show that pilots tend to have lower trait EI scores, indicating less confidence and reliance on their emotional world, with all the advantages and disadvantages this might entail, since high scores are not considered universally adaptive and desirable in trait EI theory (e.g.,^44^). Although exploratory, these findings highlight promising avenues for future trait EI research within the broader sector of international aviation. Such research will help practitioners identify new opportunities in pilot training and organizational culture, the better to equip pilots for aviation duty, ultimately leading to improved safety, performance, and all-around satisfaction.

## Data Availability

The datasets generated and/or analyzed during the current study are not publicly available because the authors wish to monitor usage by third parties, but they are available from the corresponding author on reasonable request.
